# Epidemiological Characteristics and Spatiotemporal Analysis of Acute Hemorrhagic Conjunctivitis from 2004 to 2018 in Chongqing, China

**DOI:** 10.1038/s41598-020-66467-y

**Published:** 2020-06-09

**Authors:** Dan Jing, Han Zhao, Rong Ou, Hua Zhu, Ling Hu, Mohan Giri, Mengliang Ye

**Affiliations:** 1 Department of Health Statistics and Information Management, College of Public Health and Management, Chongqing Medical University, Chongqing, 400016 P.R. China; 2 Chongqing Municipal Center for Disease Control and Prevention, Chongqing, 400042 P.R. China; 3 Library of Chongqing Medical University, Chongqing, 400016 P.R. China; 4 Departments of Respiratory Medicine, The First Affiliated Hospital of Chongqing Medical University, Chongqing, 400016 P.R. China

**Keywords:** Conjunctival diseases, Viral infection

## Abstract

Chongqing is one of the five provinces in China that has the highest incidence of acute hemorrhagic conjunctivitis (AHC). Data of AHC cases from 2004 to 2018 were obtained from National Notifiable Diseases Reporting Information System (NNDRIS). Descriptive statistical methods were used to analyze the epidemiological characteristics; incidence maps were used to reflect incidence trends in each district; spatial autocorrelation was used to identify hotspot regions and spatiotemporal patterns of AHC outbreaks; spatiotemporal scan were conducted to identify AHC clusters. A total of 30,686 cases were reported with an annual incidence of 7.04 per 100,000. The incidence rates were high in 2007 and 2014, and large epidemics were observed in 2010 with the seasonal peak in September. Individuals aged 10–19 years, males, students and farmers were the prime high-risk groups. Except for 2012 and 2013, the spatial distribution of AHC did not exhibit significant global spatial autocorrelation. Local indicators of spatial association showed that the high-risk regions are Chengkou and Wuxi. The spatiotemporal scan indicated that all clusters occurred in September 2010, and the high-incidence clusters were mainly distributed in the northeast of Chongqing. The results could assist public health agencies to consider effective preventive measures based on epidemiological factors and spatiotemporal clusters in different regions.

## Introduction

Acute hemorrhagic conjunctivitis (AHC) is a highly infectious viral disease caused by enterovirus 70 (EV70), coxsackievirus A24 variant (CA24v), or adenoviruses^1,2^. It is characterized by rapid onset of symptoms, quick dissemination and short incubation period^3^. The main manifestations of AHC are bilateral eye pain, eyelid swelling, conjunctival congestion, keratitis, foreign body sensation and increased ocular secretions^4^. Generally, the majority of patients exhibit obvious symptoms but good prognosis. A small number of patients will rapidly develop systemic symptoms such as fever, fatigue and limb pain. A few patients develop serious complications and fatal infections^5^. Since it was first reported in Ghana in 1969^6^, AHC has seen several periodic outbreaks around the world^7^, mainly in Asia, Africa and Latin America^4^. The first outbreak of AHC in China was reported in Hong Kong in 1971^8^. At present, it has spread to all provinces and cities, including some remote areas^9^. From 2005 to 2012, the reported incidence of AHC in China was 4.12 per 100,000, and Guangxi, Guangdong, Chongqing, Sichuan and Hubei were ranked as the top 5 incidence provinces^10^. Currently, AHC is still an important public health problem in China^11^, which is worth exploring and studying.

Most of the previous studies on AHC focused on the epidemiological and etiological characteristics^12^. However, the research on the spatial and temporal characteristics of AHC was still scarce. The geographical information system (GIS) has been widely used to analyze the spatiotemporal characteristics of diseases, helping to monitor and prevent infectious diseases with targeted strategies^13^.

In this study, we conducted a full scope analysis of the epidemiological and spatiotemporal characteristics of AHC in Chongqing from January 2004 to December 2018 so as to provide scientific basis for formulating policies and allocating health resources.

## Results

### Epidemiological characteristics

**Figure 1:**
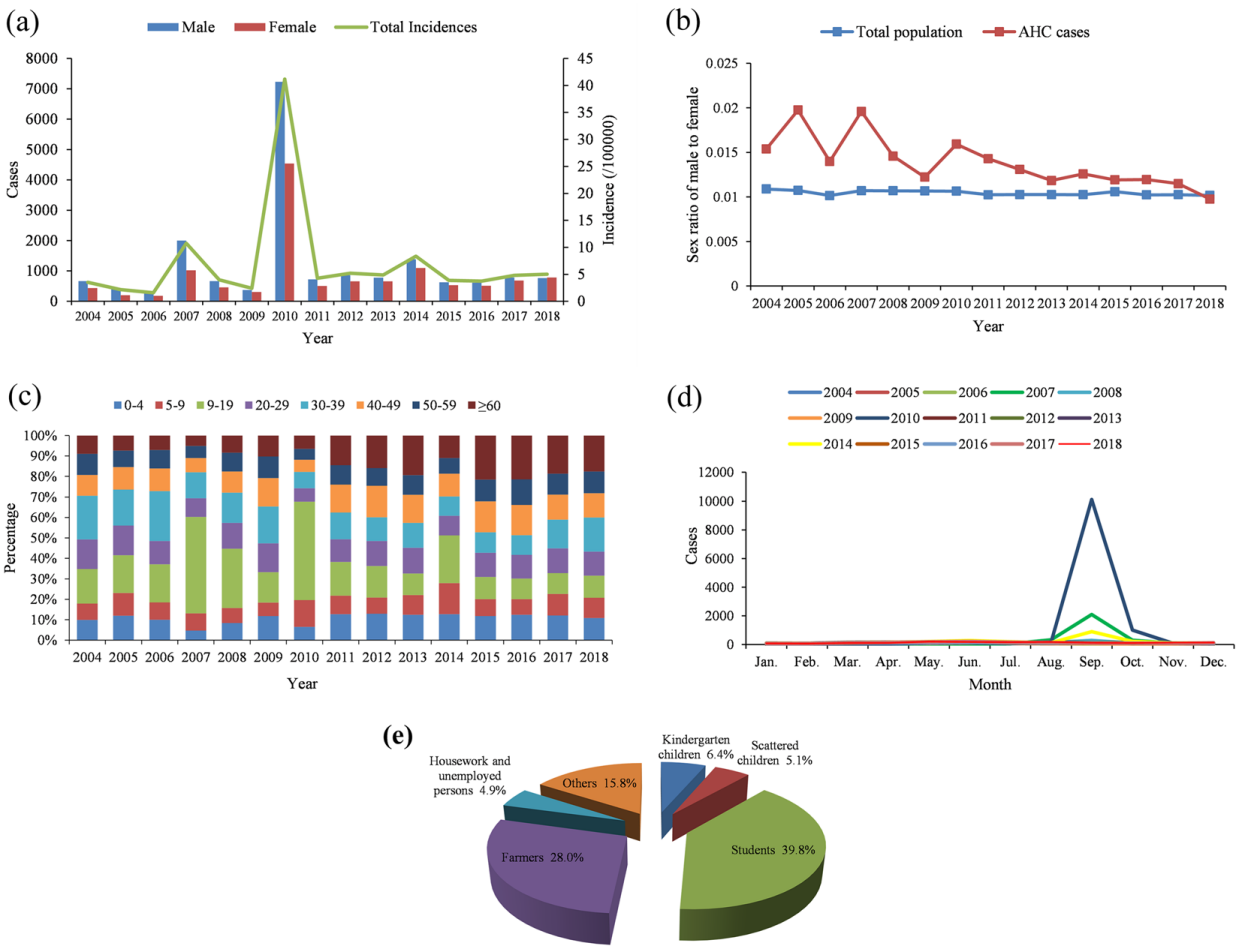
Epidemiological characteristics of AHC in Chongqing from 2004 to 2018. (**a**) The annual case number of AHC across different genders and the annual incidence of total AHC cases. (**b**) The annual male-to-female ratio of AHC cases and total population. (**c**) The age distribution of AHC cases. (**d**) The monthly distribution of AHC cases. (**e**) Occupation distribution of AHC cases.

A total of 30,686 cases of AHC were reported in Chongqing from 2004 to 2018, with an annual reported incidence of 7.04 per 100,000 (ranging from 1.57 per 100,000 in 2006 to 41.17 per 100,000 in 2010). The incidence rate reached a small peak in 2007 (10.75 per 100,000) and 2014 (8.36 per 100,000), and showed a significant outbreak trend in 2010 (41.17 per 100,000), however, the incidence rates were lower in other years. There were 18,121 male cases and 12,565 female cases and no deaths were reported in the 15 years. The average male-to-female ratio was 1.44:1, which is higher than that of Chongqing’s total population. In terms of age composition, patients aged 10–19 years old were the most affected group (31.34%). With regard to the occupation classification, the top five AHC cases were students (39.8%), farmers (28.0%), kindergarten children (6.4%), scattered children (children who do not reach the age of 3 years old to enroll in kindergarten or are taken care of by their family members) (5.1%), housework and unemployed persons (4.9%). The prevalence of AHC had obviously seasonality, with the primary peak period occurred in September, as shown in Fig. 1.

### Incidence maps

**Figure 2:**
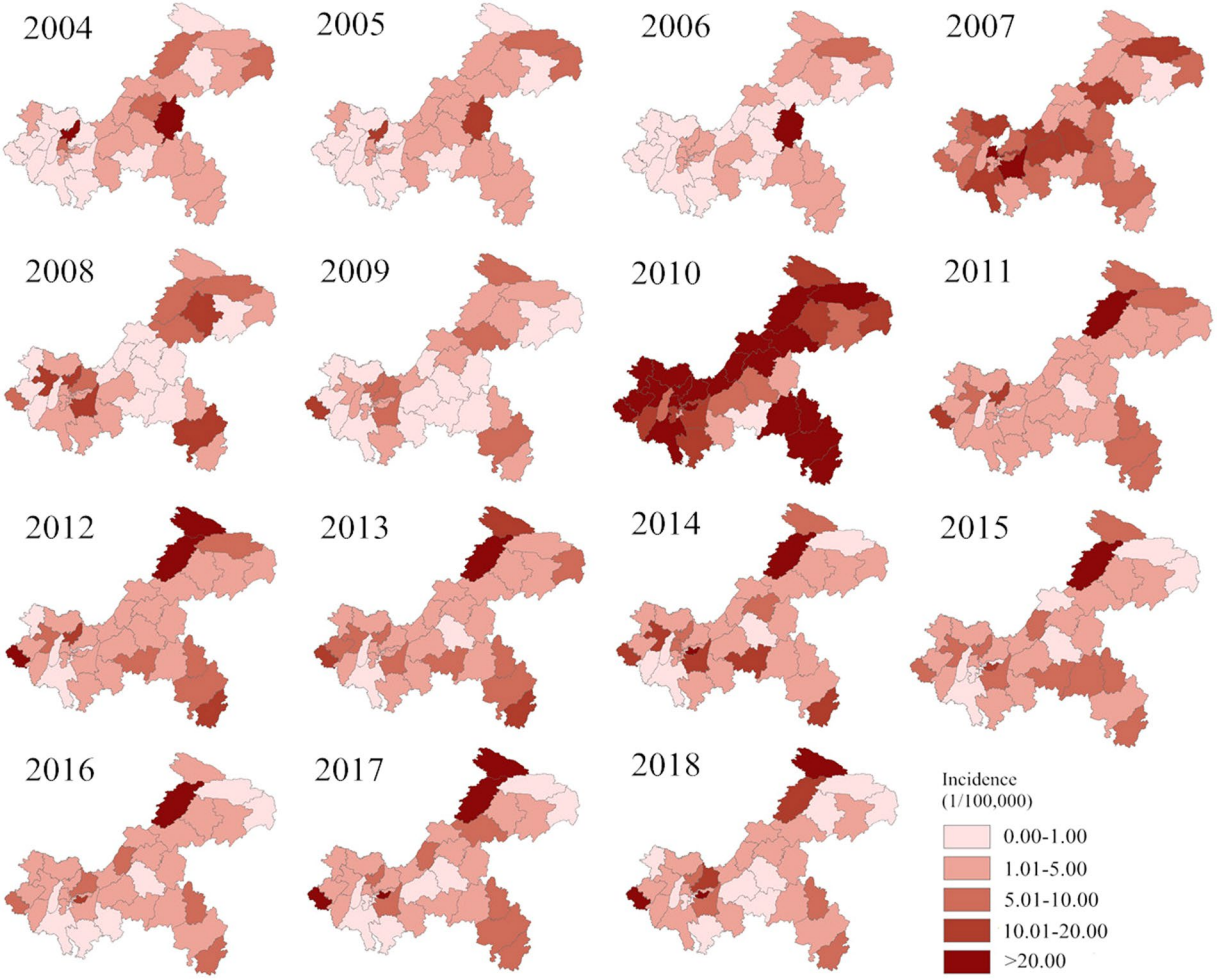
Annual incidence maps of AHC in Chongqing from 2004 to 2018. Notes: These maps generated by ArcGIS software (version 10.2 ESRI, Redlands, CA, USA, http://www.esri.com/software/arcgis/arcgis-for -desktop).

The annual incidence of AHC was mapped at the county level in Chongqing from 2004 to 2018 (Fig. 2). The high-incidence area of AHC was the Shizhu from 2004 to 2006, and gradually moved to the northeast, midwest and southeast in Chongqing from 2007 to 2009. A large outbreak of AHC was observed in 2010. The top 10 districts in terms of annual incidence were Wanzhou (59.60 per 100,000), Shapingba (52.79 per 100,000), Beibei (68.00 per 100,000), Yubei (61.85 per 100,000), Qianjiang (50.13 per 100,000), Changshou (118.35 per 100,000), Rongchang (130.82 per 100,000), Liangping (63.28 per 100,000), Dianjiang (61.54 per 100,000) and Kaixian (191.66 per 100,000). The geographical distribution was more heterogeneous from 2011 to 2018, and the high-incidence areas gradually transferred to Kaixian and Chengkou.

### Spatial autocorrelation analysis

**Figure 3:**
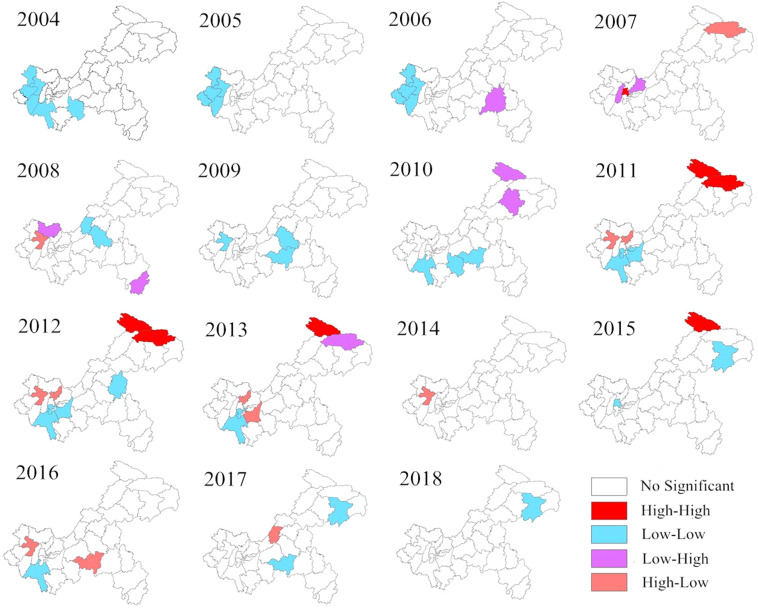
Annual local spatial autocorrelation of AHC in Chongqing from 2004 to 2018. GeoDa software (version 1.10, Spatial Analysis Laboratory, Urbana, IL, USA, https://geodacenter.github.io/download.html) was used to conduct the spatial autocorrelation analysis. These maps generated by ArcGIS software (version 10.2 ESRI, Redlands, CA, USA, http://www.esri.com/software/arcgis/arcgis-for-desktop).

Except for 2012 (Moran’s *I* = 0.2442, *p* = 0.015) and 2013 (Moran’s *I* = 0.1661, *p* = 0.039), the global spatial autocorrelation of AHC incidence in other years did not exhibit significant global correlation, the results are shown in Table 1. According to the yearly LISA cluster maps of AHC incidence, the local autocorrelation detected 7 high-high, 40 low-low, 8 low-high, and 12 high-low clusters. The high-high clusters were mainly observed in Chengkou and Wuxi. Except for 2011, 2012, 2013 and 2015, no hotspots were found in other years, as shown in Fig. 3.

**Table 1:** The global spatial autocorrelation of AHC in Chongqing from 2004 to 2018.

Year	Moran’s *I*	Z-Score	*p*-Value	Mean	SD
2004	−0.0195	0.0900	0.351	−0.0262	0.0751
2005	0.0039	0.3098	0.335	−0.0265	0.0980
2006	0.0039	0.3083	0.334	−0.0265	0.0985
2007	0.0329	0.7923	0.200	−0.0270	0.0757
2008	−0.1332	−0.9786	0.160	−0.0330	0.1024
2009	−0.0184	0.0854	0.431	−0.0273	0.1045
2010	0.0164	0.4681	0.285	−0.0298	0.0986
2011	0.1338	1.6920	0.057	−0.0273	0.0952
2012	0.2442	2.8894	0.015	−0.0279	0.0942
2013	0.1661	2.0276	0.039	−0.0238	0.0937
2014	−0.0662	−0.5541	0.307	−0.0274	0.0699
2015	−0.0293	−0.0697	0.483	−0.0235	0.0830
2016	−0.0625	−0.4053	0.387	−0.0227	0.0982
2017	0.1325	1.5177	0.074	−0.0237	0.1029
2018	0.0233	0.4458	0.292	−0.0246	0.1075

### Temporal and space-time cluster analysis

**Figure 4:**
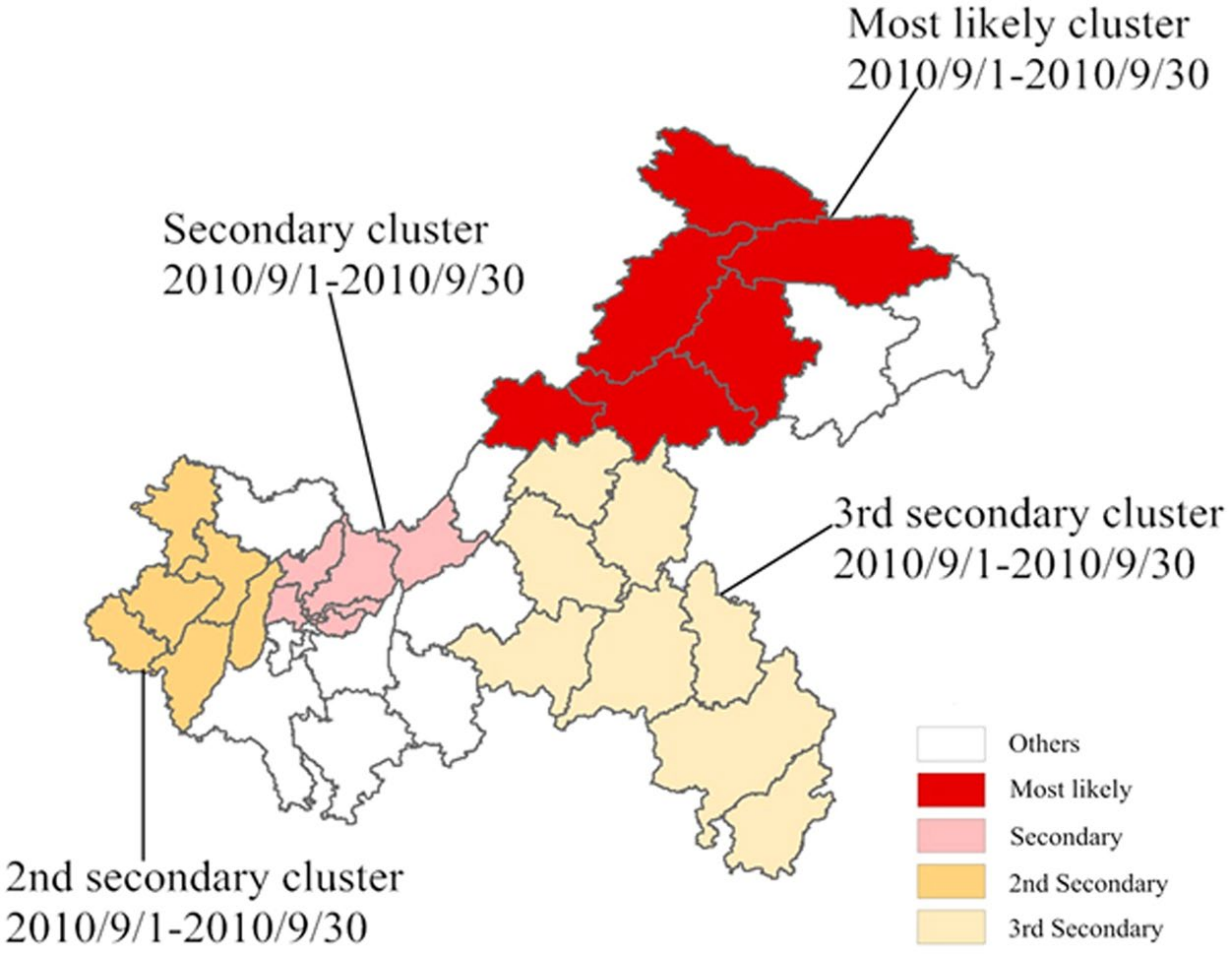
Spatiotemporal clusters of AHC cases in Chongqing from 2004 to 2018. The spatiotemporal clusters were detected by SaTScan software (version 9.5 Martin Kulldorff, National Cancer Institute, Bethesda, MD, USA, https://www.satscan.org/). These maps generated by ArcGIS software (version 10.2 ESRI, Redlands, CA, USA, http://www.esri.com/software/arcgis/arcgis-for-desktop).

The results of the purely temporal scan analysis showed that the time aggregation frame was from 2010/9/1 to 2010/9/30. Space-time scan analysis showed that four AHC clusters were found in 26 counties in Chongqing from 2004 to 2018. The most likely cluster were located in Kaixian, Yunyang, Wanzhou, Chengkou, Liangping and Wuxi, which were mainly found in the northeast part of Chongqing from 2010/9/1 to 2010/9/30 (*LLR* = 15776.99, *p* < 0.001). Other clusters (the secondary cluster, the 2nd secondary cluster and the 3rd secondary cluster) were located in the west, midwest and southeast regions of Chongqing, which were recorded from 2010/9/1 to 2010/9/30, as shown in Table 2 and Fig. 4.

**Table 2:** The cluster results of space-time scan for AHC cases in Chongqing from 2004 to 2018.

Cluster type	Time frame	Counties (n)	Radius (km)	Observed cases	Expected cases	*LLR*	*RR*	*p*-Value
Most likely	2010/9/1–2010/9/30	6	95.67	4020	31.50	15776.99	146.75	*p* < 0.001
Secondary	2010/9/1–2010/9/30	7	42.00	2264	27.18	7859.62	89.87	*p* < 0.001
2nd Secondary	2010/9/1–2010/9/30	6	80.98	1405	26.09	4253.47	56.40	*p* < 0.001
3^rd^ Secondary	2010/9/1–2010/9/30	7	125.95	750	25.42	1822.53	30.22	*p* < 0.001

## Discussion

During the 15-year study period, the incidence of AHC in Chongqing was relatively stable except for 2007, 2010 and 2014. The reason may be that AHC has a peak incidence every 3–4 years^14^. Frequent flooding may have contributed to the outbreak of AHC in Chongqing in 2010^15^. In the same year, large-scale epidemics also occurred in Guangzhou, Guangxi, Beijing, Hainan, Zhejiang and Shandong provinces in China^16,17^. Prior research showed that water sources, transportation and living infrastructure would be damaged and polluted after floods. Moreover, the decline of sanitary conditions and the poor psychological and physical state of the people afflicted by the natural disaster tend to lead to the outbreak and epidemic of AHC^18^. CA24v was found to be the pathogen causing the outbreak in 2010 and belongs to genotype IV^19^.

The incidence of this disease occurs all year round, and the month of high incidence was in September, which may be related to the hot and humid summer and autumn in Chongqing, and providing a suitable environment for the survival and transmission of AHC pathogens^20,21^. At this time, students were enrolled in school, resulting in the crowding of individuals. The students infected with the disease were not detected or treated in time, leading to the outbreak of AHC in the school within a short period after enrollment. Individuals aged 10–19 years old, males, students and farmers were more likely to suffer from AHC than other groups, which was consistent with the research results in Yichang and Jinan^22,23^. AHC is prone to spread in densely populated areas with poor medical and health conditions. Farmers lack adequate health knowledge and good health habits, and live in places with inadequate medical and health facilities. The infection source was not been effectively controlled and isolated, which is an important cause of the outbreak^24,25^. Over the years, the total number of male cases was higher than that of female cases^26^. Moreover, there was a significant gender difference was observed in the incidence of AHC among individuals aged below 20 years, which may be due to the difference in lifestyle and hygiene habits. Males are hyperactive or have poor hygiene awareness and are engaged in different types of occupational labor^27,28^.

The high-risk areas detected by LISA map were mainly concentrated in some suburbs, such as Chengkou and Wuxi. The clustering time determined by temporal and space-time cluster analysis was consistent with the observed years of high incidence rate. The high-risk areas were not only the marginal districts (counties) in the northeast, but also the main urban districts (counties) and their adjacent area in the central and western regions.

AHC has no specific preventive measures. Public health authorities should strengthen surveillance of the epidemic among males, students, farmers and other high-risk groups, as well as high-risk areas in the northeast, central and western regions, to improve the availability and equality of medical and health resources in all regions^29^. In the seasons with high incidence of AHC, schools and other key public places should be mainly monitored. Strengthening health education and improving residents’ health literacy are the key to prevent and control AHC outbreaks^30^.

This study is the first time to monitor AHC epidemic data in Chongqing city by spatio-temporal analysis, and further explored the epidemic and spatial distribution characteristics of AHC, providing theoretical basis for the prevention and control of AHC in Chongqing. However, this study also has some shortcomings. Firstly, due to different prevention and control strategies and medical levels in different areas, the number of reported AHC cases may be underreported, and the quality of data may vary from region to region. Secondly, relevant meteorological, socio-economic factors and detailed patient onset information were not included in this study, which failed to further explain the causal relationship of AHC onset.

## Conclusion

The study found that except for the high incidence in 2007, 2014 and the large outbreak in 2010, AHC incidence in Chongqing was generally stable in the past 15 years. Compared with other groups, teenagers aged 10–19 years old, males, students, and farmers were more likely to suffer from AHC, the peak period of which was in September every year. Suburban areas in the northeast and urban areas in the midwest should be taken as the key monitoring areas. The results provide evidence for health authorities to take effective interventions, such as protection and health education of high-risk groups, real-time monitoring of high-risk areas, and rational allocation of medical resources.

## Materials and Methods

### Profile of chongqing city

**Figure 5:**
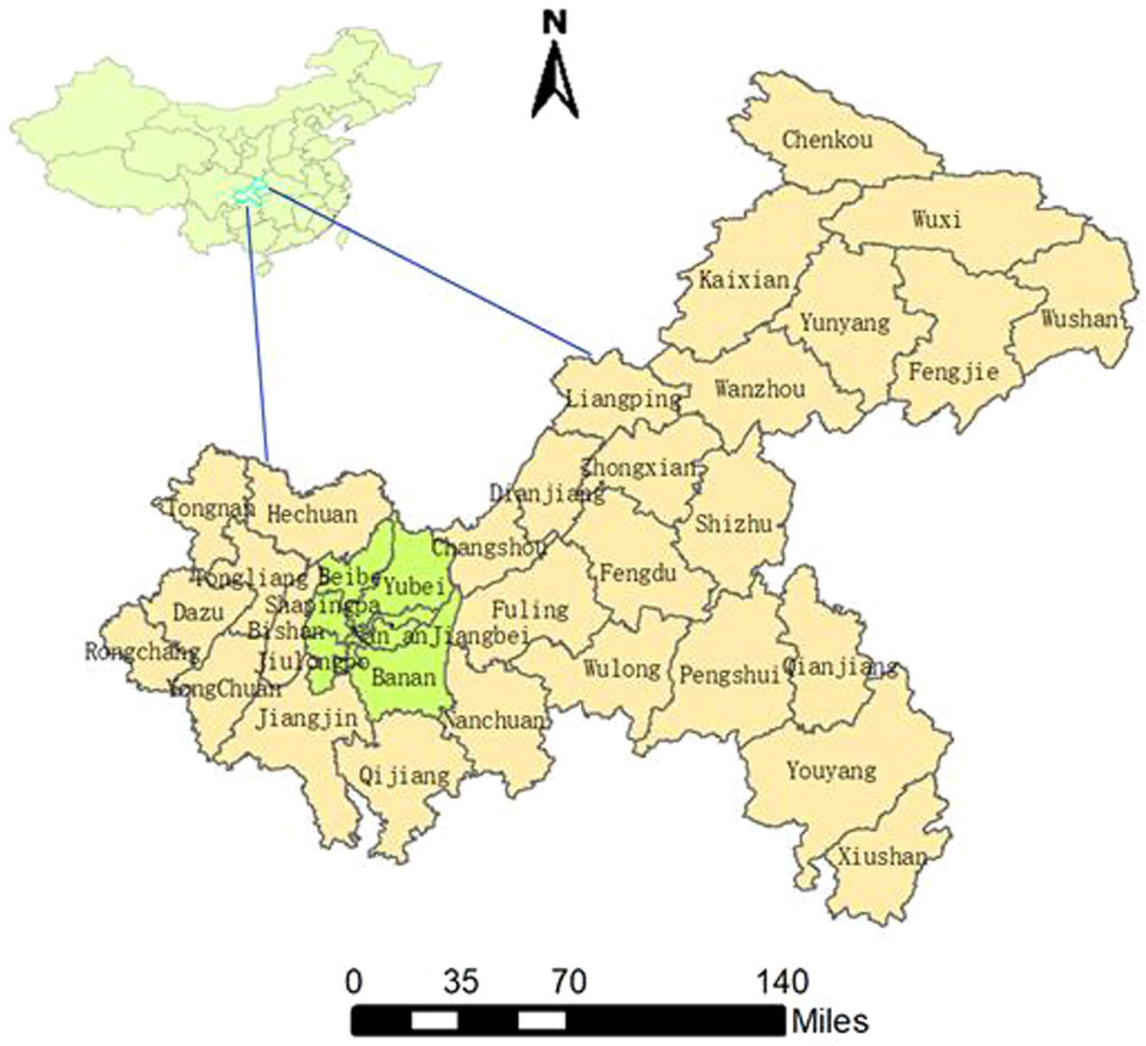
The geographical location and administrative division of Chongqing at the county level in China. The cyan part represents the nine main urban districts. China and Chongqing maps were generated by ArcGIS software (version 10.2 ESRI, Redlands, CA, USA, http://www.esri.com/software/arcgis/arcgis-for-desktop).

Chongqing (28°10′–32°13′ N, 105°11′–110°11′E) is an important economic city in southwest China. It has a subtropical monsoon climate and dominated by hills and mountains. Chongqing is a high-humidity area in China, with an average annual temperature of 16~18 °C and an average relative humidity of 70%~80%^31^. Chongqing covers an area of 82,403 km^2^, and the resident population was approximately 31.01 million by the end of 2018. Its administrative district is divided into 23 municipal districts, 11 counties and 4 autonomous counties (Fig. 5).

### Data collection

In 2004, NNDRIS was established in China^32^. As a class C infectious disease in mainland China^33^, all patients diagnosed with AHC should be reported to the system within 24 hours by local physicians. Data of AHC cases in Chongqing from 2004 to 2018 included the year of report, patient gender, age, address and occupation. Population data were provided by the Chongqing Information System for Diseases Control and Prevention, and vector maps of China and Chongqing were obtained from the National Basic Geographic Information System.

### Spatial autocorrelation analysis

Spatial autocorrelation analysis is a spatial method used to describe the spatial autocorrelation association based on the locations of study regions^34,35^, global and local Moran’s *I* were used to identify whether the significant spatial autocorrelation regions of AHC in Chongqing. In general, they are separated into the “global” and “local” categories.

#### Global indicators of spatial association (GISA)

GISA with the indicator of Moran’s *I* statistic reflected the spatial associations of AHC incidence in a global area. The significance of Moran’s *I* was validated by Monte Carlo tests with *Z* statistics and the *p-*values. The Moran’s *I* ranged from −1 to 1. If *I* > 0, it indicates that the distribution has a positive autocorrelation. If *I* < 0, it means that the distribution has a global negative autocorrelation. If *I* = 0, the spatial distribution is random^36,37^. The *p*-values (*p* < 0.05) and Z-scores (|Z | < 1.96) were utilized to determine the statistical significance of Moran’s *I*.

#### Local indicators of spatial association (LISA)

By using local indicators of spatial association (LISA) map, we clustered in the significant spatial autocorrelation regions and displayed them in the form of significant and clustering graphs^38,39^. Four spatial patterns could be seen intuitively in LISA maps^40^, high-high (high-incidence regions surrounded by high-incidence regions, which are highly epidemical regions), low-low (low-incidence regions surrounded by low-incidence regions, which are lowly epidemical regions), high-low (high-incidence regions surrounded by low-incidence regions) and low-high (low-incidence regions surrounded by high-incidence regions)^41,42^.

### Scan statistic

SaTScan software was used to identify the possible spatiotemporal clusters and clustering time^43^. In this study, the geographical clusters were calculated by Kulldorff’s method of retrospective space-time scan statistic, and a discrete Poisson model was selected as the probability model to estimate the high-incidence area of AHC at the county level^44^. The space-time scan statistic is defined by a circular (or elliptic) window, and each window is in turn centered on each geographic region of the study area^45^. The center and radius of the bottom and the height are constantly changing. The maximum spatial cluster radius was designated as 20% of the high-risk population in the spatial window, and the maximum temporal cluster radius was designated as 50% of the study period in the time window. The Log likelihood ratio (*LLR*) was obtained based on the actual and theoretical cases inside and outside the window. The most likely cluster was the window that had the maximum *LLR*^46^, and the other windows with the statistically significant *LLR* were the secondary clusters, which were ranked in order of the *LLR* value. The number of Monte Carlo simulations was set to the default value of 999, and the *p-*value was obtained by Monte Carlo hypothesis test.

### Statistical software

In this study, descriptive statistical methods were used to investigate the population distribution, occupational composition and seasonal characteristics of AHC cases. GeoDa software (version 1.10, Spatial Analysis Laboratory, Urbana, IL, USA) was used to conduct the spatial autocorrelation analysis. The spatiotemporal clusters were detected by SaTScan software (version 9.5 Martin Kulldorff, National Cancer Institute, Bethesda, MD, USA). ArcGIS software (version 10.2 ESRI, Redlands, CA, USA) was used for mapping and visualization analysis. All results were considered statistically significant when the *p* < 0.05 for both sides.
